# Identification and Characterization of FAM124B as a Novel Component of a CHD7 and CHD8 Containing Complex

**DOI:** 10.1371/journal.pone.0052640

**Published:** 2012-12-21

**Authors:** Tserendulam Batsukh, Yvonne Schulz, Stephan Wolf, Tamara I. Rabe, Thomas Oellerich, Henning Urlaub, Inga-Marie Schaefer, Silke Pauli

**Affiliations:** 1 Institute of Human Genetics, University Medical Center, Göttingen, Germany; 2 Department of Molecular Cell Biology, Max Planck Institute for Biophysical Chemistry, Göttingen, Germany; 3 Bioanalytical Mass Spectrometry Group, Max Planck Institute for Biophysical Chemistry, Göttingen, Germany; 4 Bioanalytics, Department of Clinical Chemistry, University Medical Center, Göttingen, Germany; 5 Department of Pathology, University Medical Center, Göttingen, Germany; Leibniz Institute for Age Research - Fritz Lipmann Institute (FLI), Germany

## Abstract

**Background:**

Mutations in the chromodomain helicase DNA binding protein 7 gene (CHD7) lead to CHARGE syndrome, an autosomal dominant multiple malformation disorder. Proteins involved in chromatin remodeling typically act in multiprotein complexes. We previously demonstrated that a part of human CHD7 interacts with a part of human CHD8, another chromodomain helicase DNA binding protein presumably being involved in the pathogenesis of neurodevelopmental (NDD) and autism spectrum disorders (ASD). Because identification of novel CHD7 and CHD8 interacting partners will provide further insights into the pathogenesis of CHARGE syndrome and ASD/NDD, we searched for additional associated polypeptides using the method of stable isotope labeling by amino acids in cell culture (SILAC) in combination with mass spectrometry.

**Principle findings:**

The hitherto uncharacterized FAM124B (Family with sequence similarity 124B) was identified as a potential interaction partner of both CHD7 and CHD8. We confirmed the result by co-immunoprecipitation studies and showed a direct binding to the CHD8 part by direct yeast two hybrid experiments. Furthermore, we characterized FAM124B as a mainly nuclear localized protein with a widespread expression in embryonic and adult mouse tissues.

**Conclusion:**

Our results demonstrate that FAM124B is a potential interacting partner of a CHD7 and CHD8 containing complex. From the overlapping expression pattern between Chd7 and Fam124B at murine embryonic day E12.5 and the high expression of Fam124B in the developing mouse brain, we conclude that Fam124B is a novel protein possibly involved in the pathogenesis of CHARGE syndrome and neurodevelopmental disorders.

## Introduction

In humans, CHD7 (NM _017780) is one of nine members of the chromodomain helicase DNA binding domain (CHD) family that plays a role in controlling gene expression by ATP-dependent chromatin remodeling. Mutations in the *CHD7* gene are the major cause of CHARGE syndrome (OMIM 214800), an autosomal dominant congenital malformation disorder characterized by the combination of eye, ear, craniofacial structure, and heart defects [1]–[6]. However, in 5–10% of patients with a typical presentation of CHARGE syndrome and in 40–60% of patients with an atypical presentation the underlying cause of the symptoms remains unclear [7]. For other autosomal dominant disorders, e.g. Noonan syndrome, a genetic heterogeneity is known, wherein mutations in different genes lead to a similar phenotype. Therefore, we hypothesize that in CHARGE syndrome, besides mutations in *CHD7,* mutations in one or more additional and hitherto unknown genes are involved in the pathogenesis of this disease.

Proteins involved in chromatin remodeling are typically found in multiprotein complexes. In recent and earlier studies different CHD7 interacting partners have been described [8]–[13]. In human neural crest-like cells CHD7 was shown to be associated with components of the BAF- (Brahma associated factor complex) and PBAF - complexes (Polybromo containing complex) [10]. Both belong to the SWI/SNF-family of ATP-dependent chromatin remodeling complexes and can act as transcriptional activators or repressors [14]. In murine embryonic stem (ES) cells co-localization between Chd7 and the proteins p300, Oct4, Sox2, Nanog, Smad1 and Stat3 at enhancer elements was shown [12] leading to the hypothesis that these proteins are cofactors in enhancer promoter interactions [12]. CHD7 was also found to be associated with treacle, the protein that is involved in the pathogenesis of Treacher Collins syndrome [13]. These studies demonstrate that there are numerous CHD7 interacting partners, leading to the suggestion that there are cell type specific compositions of CHD7 containing complexes and that the subunits may change during development [7].

Recently, we demonstrated that a part of the human CHD7 protein interacts with a part of the CHD8 protein, another CHD family member. Studies in *Drosophila melanogaster* demonstrated that *kismet* is the only gene related to the human subgroup III members (CHD6-CHD9). *Kismet* has a functional role in transcriptional regulation by promoting early elongation by RNA Polymerase II as well as by recruiting the histone methyltransferases ASH1 and TRX to chromatin [15]. Rodriguez-Paredes et al. suggested that in mammals the function of *kismet* is overtaken by several subgroup III members (CHD6-CHD9) [16] and we hypothesized that CHD7 and CHD8 build a core component of a complex with similar functions such as *kismet* [11]. CHD8 was found to be associated with the WAR complex [17]. This complex includes WDR5, ASH2L and RbBP5 (WAR) and is known as a subcomplex of mixed lineage leukemia (MLL) complexes, the *Drosophila* homolog to TRX complexes. The MLL complexes act as histone H3 Lys-4 methyltransferases [18].

Furthermore, CHD8 directly binds beta-catenin and negatively regulates beta-catenin-targeted gene expression [17]. Microdeletions, chromosomal rearrangements disrupting *CHD8* as well as de novo missense and nonsense mutations in the *CHD8* gene were described in autism spectrum (ASD) and in neurodevelopmental (NDD) disorder patients, indicating that alterations in *CHD8* can contribute to ASD and NDD [19]–[22].

Identification of novel CHD7 and CHD8 interacting partners will provide further insights into the pathogenesis of CHARGE syndrome and ASD/NDD. Therefore, we tried to detect new binding partners by using the method of stable isotope labeling by amino acids in cell culture (SILAC) in combination with mass spectrometry. We identified FAM124B (Family with sequence similarity 124B) as a potential interaction partner of both CHD7 and CHD8. Additionally, we confirmed the interaction by co-immunoprecipitation and performed direct yeast two hybrid experiments. Furthermore, we examined the intracellular localization and tissue specific expression of Fam124B during mouse embryogenesis and in adult mouse tissues.

## Results

### Identification of FAM124B as Part of the CHD7 and CHD8 Interactomes

**Figure 1:**
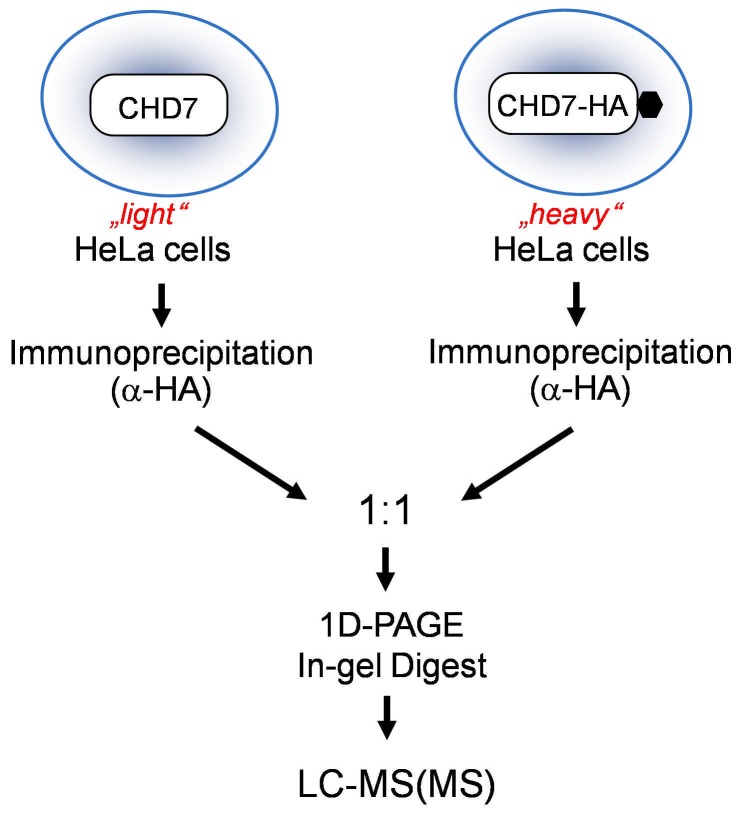
Schematic overview of the SILAC approach. “Heavy”-labeled cells were co-transfected with the plasmids CHD7-CR1-3-pCMV-HA (containing amino acids 1593-2178, NP_060250.2, in fusion with an HA-tag) and CHD8-pCMV-cmyc (spanning amino acids 1789–2302, NP_065971.2, in fusion with a cmyc-tag). The CHD7 part was purified by anti-HA immunoprecipitation. As a negative control, the same immunoprecipitation was performed in lysates of non-transfected “Light”-labeled HeLa cells. Purified proteins from both cell cultures were pooled in equimolar amounts and in-gel digested, followed by liquid-chromatography (LC)-coupled tandem mass spectrometry.

In order to identify novel CHD7 and CHD8 interaction partners we applied stable isotope labeling by amino acids in cell culture (SILAC) in combination with mass spectrometry [23], [24] (Figure 1). To achieve differential isotope labeling of HeLa cells, one cell batch was cultured in the presence of lysine and arginine containing light (L) isotopes of carbon and nitrogen (^12^C and ^14^N, l-lysine and l-arginine) while the other batch was cultured in the presence of lysine and arginine containing heavy (H) isotopes of carbon and nitrogen (^13^C_6_^15^N_2_-lysine and ^13^C_6_^15^N_4_-arginine). Accordingly, the two culture conditions confer distinct molecular masses on the cellular proteins and in this way proteins derived from SILAC-labeled cells can be distinguished and thus attributed to the L- or H-labeled cell batch by mass spectrometry. For elucidation of the CHD7- or CHD8 interactomes, the H-labeled cells were firstly co-transfected with the plasmids CHD7-CR1-3-pCMV-HA (containing amino acids 1593-2178, NP_060250.2, in fusion with an HA-tag) and CHD8-pCMV-cmyc (spanning amino acids 1789–2302, NP_065971.2, in fusion with an cmyc-tag). 24 hours post transfection, expression of the tagged proteins was confirmed by western blotting. Subsequently, the respective H-labeled cells were lysed and the CHD7 part was purified by anti-HA immunoprecipitation. As a negative control the same immunoprecipitation was performed in lysates of non-transfected L-labeled HeLa cells. The purified proteins from both the H- and L-states were then pooled in equimolar amounts and subsequently digested with the endoproteinase trypsin. Derived peptides were identified by liquid-chromatography (LC)-coupled tandem mass spectrometry (MS/MS), allocated to the corresponding proteins by database search and finally quantified using the MaxQuant software. An at least five-fold enrichment of heavy versus light peptides was considered to mark proteins that were specifically co-purified with the HA-tagged CHD7 part. The same experimental workflow was also performed with inverse labeling. In at least three biological replicates FAM124B was co-purified with CHD7 and CHD8, identifying this so far uncharacterized protein as a novel effector of the CHD7/8 interactome (Table S1–S3).

### Full Length Cloning and Transcript Analysis of FAM124B

**Figure 2:**
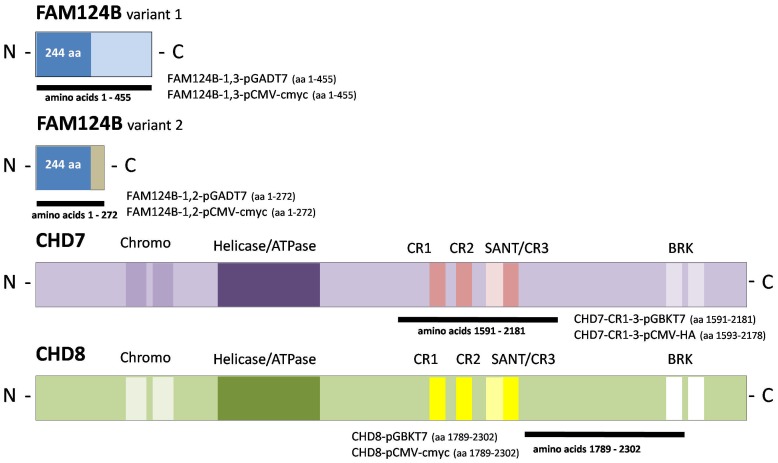
Schematic overview of FAM124B, CHD7 and CHD8 and the constructs used. In humans, there are two transcript variants of FAM124B. Transcript variant 1 codes for a protein with 455 amino acids (NP_001116251.1), while transcript variant 2 leads to a shorter protein product with 272 amino acids (NP_079061.2). Both variants have the first 244 amino acids in common. All used FAM124B variant 1 and 2 constructs are full length constructs. Bioinformatic analysis of the amino acid sequence of FAM124B failed to identify any known functional domain, while CHD7 and CHD8 consist of two N-terminal chromodomains (Chromo), followed by a SWI2/SNF2-like ATPase/helicase domain (Helicase/ATPase), three conserved regions (CR1-3), a SANT domain and two BRK domains. The black lines mark the regions cloned into the Yeast two hybrid (pGBKT7, pGADT7) and Co-IP (pCMV-HA, pCMV-cmyc) vectors.

Information of the genomic and cDNA structure of FAM124B was obtained from NCBI database. In humans, two transcript variants are described. Transcript variant 1 contains two exons with the ATG in exon one and the stop codon in exon 2 resulting in a protein with 455 amino acids (NP_001116251.1). Transcript variant 2 contains an alternate exon with an in-frame stop codon leading to a shorter protein product with 272 amino acids (NP_079061.2) (Figure 2). In mice, one transcript homologe to the human transcript variant 1 containing 456 amino acids was described (NP_775601.1). We validated the information by full length cloning and sequencing of human and mouse cDNAs derived from HeLa cells and mouse adult brain tissue, respectively.

### FAM124B Co-immunoprecipitates with a Part of CHD7 and a Part of CHD8

**Figure 3:**
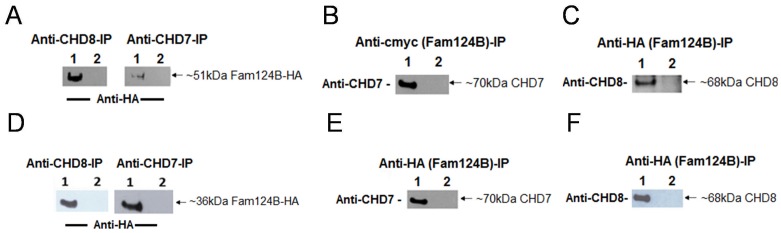
Co-immunoprecipitation of FAM124B with a part of CHD7 and CHD8. HeLa cells were co-transfected with either the CHD7-CR1-3-pCMV-HA (amino acids 1593-2178, NP_060250.2) plasmid and FAM124B-1,3-pCMV-cmyc or FAM124B-1,3-pCMV-HA (transcript variant 1, NP_001116251.1) or with CHD8-pCMV-cmyc (amino acids 1789-2302, NP_065971.2) and FAM124B-1,3-pCMV-HA (transcript variant 1, NP_001116251.1). (**A**) Using the anti-CHD8 (abcam, ab84527) or the anti-CHD7 (abcam, ab31824) antibody for precipitation, we detected with the anti-HA antibody (Roche) an approximately 51 kDa band corresponding to the estimated size of FAM124B transcript variant 1. Lane 1: co-transfected Co-IP, lane 2: untransfected HeLa cells as negative control. (**B**) Reciprocal immunoprecipitation with anti-cmyc antibody (precipitating FAM124B transcript variant 1), and detection with the anti-CHD7 antibody lead to a specific band ∼70 kDa, the estimated size for the CHD7 part fused to the HA-tag. Lane 1: co-transfected Co-IP, lane 2: untransfected HeLa cells as negative control. (**C**) Reciprocal experiment with anti-HA antibody (precipitating FAM124B transcript variant 1) and detection with the anti-CHD8 antibody detected a specific band ∼68 kDa, the estimated size for the CHD8 part fused to the cmyc-tag. Lane 1: co-transfected Co-IP, lane 2: untransfected HeLa cells as negative control. (**D, E, F**) The same experimental procedure was performed for FAM124B transcript variant 2, demonstrating a specific interaction of FAM124B transcript variant 2 with the CHD7 and CHD8 part as well. Lane 1: co-transfected Co-IP, lane 2: untransfected HeLa cells as negative control.

Co-Immunoprecipitation studies on HeLa cells were performed to confirm the CHD7-CHD8- FAM124B interaction. HeLa cells were co-transfected with either the CHD7-CR1-3-pCMV-HA (amino acids 1593-2178, NP_060250.2) plasmid and FAM124B-1,3-pCMV-cmyc (transcript variant 1, NP_001116251.1) or with CHD8-pCMV-cmyc (amino acids 1789-2302, NP_065971.2) and FAM124B-1,3-pCMV-HA (transcript variant 1, NP_001116251.1) (Figure 2). Total protein was isolated after 24 hours. Immunoprecipitation with either the anti-CHD7 (abcam, ab31824) or the anti-CHD8 antibody (abcam, ab84527) and detection with either the anti-cmyc (abcam, ab9106) or anti-HA antibody (Roche) lead to an approximately 51 kDa band corresponding to the estimated size of FAM124B transcript variant 1 (Figure 3A). Reciprocal immunoprecipitation with anti-cmyc antibody and detection with the anti-CHD7 antibody demonstrated a specific band of ∼70 kDa, the estimated size for the CHD7 part fused to the HA-tag (Figure 3B). Using the anti-HA-antibody for precipitation, we detected a ∼68 kDa band corresponding to the estimated size of the CHD8 part fused to the cmyc tag (Figure 3C) by using the anti-CHD8 antibody.

The same experimental procedure was then performed with the plasmid FAM124B-1,2-pCMV-HA (transcript variant 2, NP_079061.2). Similar to the results for the FAM124B transcript variant 1, we demonstrated an interaction with the CHD7 and CHD8 part with FAM124B transcript variant 2 (Figure 3D–F).

### FAM124B Interacts Directly with a Part of CHD8

**Figure 4:**
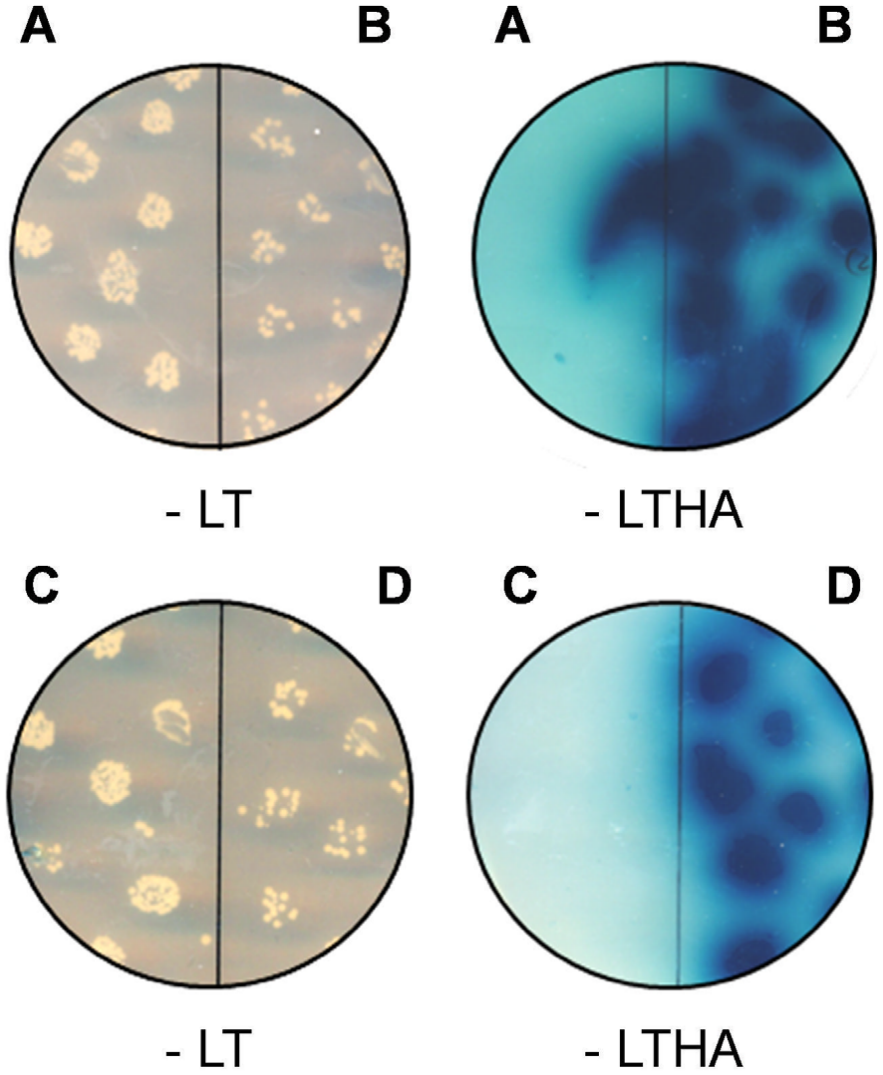
Yeast two hybrid assay. (**A**) Direct yeast two hybrid experiment with the constructs FAM124B-1,3-pGADT7 (full length transcript variant 1) and CHD7-CR1-3-pGBKT7 (amino acids 1591-2181, NP_060250.2) demonstrating no direct interaction between FAM124B transcript variant 1 and the CHD7 part, while (**B**) Direct yeast two hybrid experiment with the constructs FAM124B-1,3-pGADT7 (full length transcript variant 1) and CHD8-pGBKT7 (amino acids 1789–2302, NP_065971.2) shows a direct interaction. The same experiments were performed for FAM124B transcript variant 2. (**C**) Direct yeast two hybrid experiment with the constructs FAM124B-1,2-pGADT7 (full length transcript variant 2) and CHD7-CR1-3-pGBKT7 (amino acids 1591-2181, NP_060250.2). (**D**) Direct yeast two hybrid experiment with the constructs FAM124B-1,2-pGADT7 (full length transcript variant 2) and CHD8-pGBKT7 (amino acids 1789–2302, NP_065971.2). FAM124B transcript variant 2 interacts directly with the CHD8 part, while no direct interaction with the CHD7 part could be observed.

Y2H experiments were performed to determine a direct interaction between CHD7, CHD8 and both variants of FAM124B using the following plasmids: FAM124B-1,3-pGADT7 (full length transcript variant 1), FAM124B-1,2-pGADT7 (full length transcript variant 2), CHD7-CR1-3-pGBKT7 (amino acids 1591-2181, NP_060250.2) and CHD8-pGBKT7 (amino acids 1789–2302, NP_065971.2) (Figure 2). The yeast two hybrid experiments revealed that both transcripts of FAM124B directly interact with the CHD8 part, while no direct interaction with the CHD7 part, spanning the amino acids 1591-2181, could be observed (Figure 4). Because FAM124B transcript variants 1 and 2 have exon 1 in common, we hypothesized that the FAM124B-CHD8 interacting area is located within exon 1 of FAM124B.

### Subcellular Localization of FAM124B in HeLa Cells

**Figure 5:**
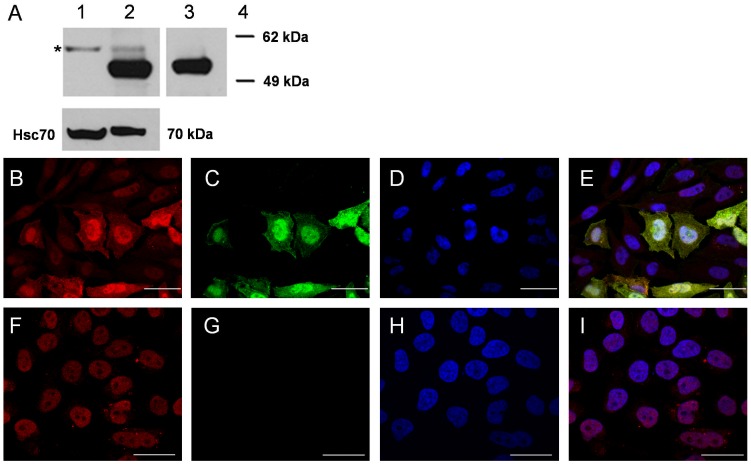
Western blot and immunocytochemistry of endogenous and overexpressed FAM124B. (**A**) Western blot analysis on protein isolated from untransfected HeLa cells (endogenous FAM124B) and HeLa cells overexpressing the FAM124B-1,3-cmyc-tag fusion protein. Lane 1: Immunoblotting of the nuclear cell fraction of untransfected HeLa cells using the FAM124B antibody. The predicted size of human endogenous FAM124B-1,3 is approximately 51 kDa. However, we observed a band of approximately 57 kDa (labeled by an asterisk). We hypothesize that this band could be endogenous FAM124B and the larger size is possibly due to posttranslational modifications of the endogenous protein. Lane 2: Immunoblotting of FAM124B-1,3-pCMV-cmyc overexpressed HeLa total cell lysate using the rabbit anti-FAM124B antibody revealed a prominent band of 51 kDa, the calculated size of human full length FAM124B variant 1 in fusion with a c-myc-tag and the band observed in untransfected HeLa cells. Lane 3: Immunoblotting of the same HeLa cell lysate as shown in lane 2 using anti-c-Myc shows the 51 kDa band corresponding to the overexpressed FAM124B. Lane 4: Marker. Protein quality was controlled using mouse anti-HSC70 producing a 70 kDa band. (**B**) Immunofluorescence staining using the rabbit anti-Fam124B antibody on HeLa cells transiently transfected with the plasmid FAM124B-1,3-pCMV-cmyc (FAM124B transcript variant 1 fused to an c-myc-tag). Due to the transient transfection not all cells overexpressed FAM124B. Transfected cells show a bright red signal in the nucleus demonstrating a nuclear distribution. The weaker signal in the cell nuclei of the untransfected surrounding cells could possibly reflect an endogenous FAM124B expression, which we detected by RT-PCR and mass spectrometry. (**C**) Immunofluorescence staining using the anti-c-Myc antibody on the same HeLa cells transiently transfected with the plasmid FAM124B-1,3-pCMV-cmyc showing a bright green signal in the nucleus of the transfected cells. No signal could be observed in the surrounding untransfected cells. (**D**) Cell nuclei were stained with DAPI (blue). (**E**) Overlay of B,C and D. (**F**) Immunofluorescence staining using the rabbit anti-Fam124B antibody on untransfected HeLa cells revealed a weak nuclear signal as it was observed in the surrounding untransfected cells (see Figure 5B). (**G**) Immunofluorescence staining using the anti-c-Myc antibody on untransfected cells reveals no signal, as expected. (**H**) Staining of cell nuclei with DAPI (**I**) overlay of F,G and H. B,C,D,E: Scale bar = 40 µm. F,G,H,I: Scale bar = 30 µm.

To determine the subcellular localization of endogenous FAM124B in HeLa cells, we performed immunofluorescence staining by using a rabbit anti-FAM124B antibody (ProteinTech). Overexpressed FAM124B is localized mainly in the nucleus (Figure 5B). To test the specificity of the polyclonal anti-FAM124B antibody, we transiently transfected HeLa cells with the plasmid FAM124B-1,3-pCMV-cmyc to overexpress cmyc epitope tagged FAM124B-1,3 (transcript variant 1, NP_001116251.1). By using the rabbit anti-FAM124B antibody, the nuclear localization of the overexpressed protein was indicated by an intense signal (Figure 5B). Immunofluorescence staining of recombinant c-myc tagged FAM124B on the same cells using the anti-c-Myc-antibody (abcam, ab9106) confirmed these results (Figure 5C). Due to the transient transfection not all cells overexpressed FAM124B. By using the anti-FAM124B antibody a weaker signal in the cell nuclei of the untransfected surrounding cells (Figure 5B) and the untransfected cells (Figure 5F) could be observed, indicating a possible endogenous FAM124B expression.

### Western Blot Analysis of Overexpressed FAM124B

Additionally, we performed western blot analysis on the nuclear cell fraction of untransfected HeLa cells (endogenous FAM124B) and FAM124B-1,3-pCMV-cmyc overexpressed HeLa total cell lysate (overexpression of c-myc epitope tagged FAM124B-1,3). Immunoblotting of the nuclear cell fraction of untransfected HeLa cells revealed a specific band of approximately 57 kDa. The predicted size of human endogenous FAM124B-1,3 is approximately 51 kDa. We hypothesize that the observed band could be endogenous FAM124B and suggested that the difference between the predicted and the observed size is due to posttranslational modifications of the endogenous FAM124B. Immunoblotting performed on FAM124B-1,3-pCMV-cmyc overexpressed HeLa total cell lysate using the anti-FAM124B antibody revealed a prominent band of 51 kDa, the estimated size of the overexpressed FAM124B variant 1 in fusion with a c-myc-tag and weaker the above described 57 kDa band. Immunoblotting using the anti-c-Myc antibody showed only the 51 kDa band, representing the overexpressed FAM124B fusion protein (Figure 5A).

### Tissue Specific Expression of Murine Fam124B

**Figure 6:**
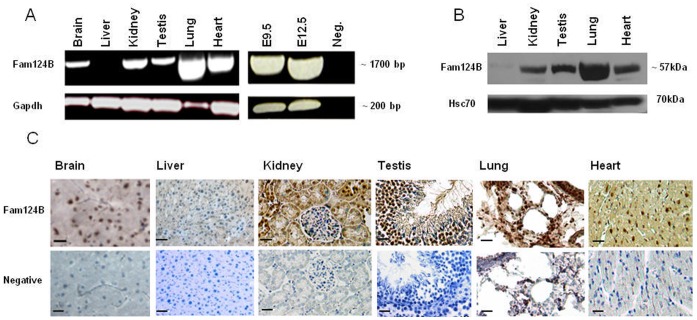
Expression pattern of murine Fam124B. (**A**) Expression pattern of murine Fam124B by semiquantitative reverse transcription polymerase chain reaction (RT-PCR) on wild type CD1 mouse tissues and E9.5 and E12.5 embryos demonstrating expression in various tissues and during development. (**B**) Western blot analysis of endogenous Fam124B on various mouse tissues using the anti-FAM124B antibody demonstrates expression in heart, testis, kidney and highest in lung. A weak signal was detected in liver. (**C**) Immunohistochemistry (IHC) performed on adult mouse tissues slightly counterstained with haematoxylin (blue) confirmed the semiquantitative RT-PCR and western blot results with high expression (brown) in lung, heart, kidney, moderate expression in brain and testis, and very low expression in liver (most liver cell nuclei are only stained with haematoxylin (blue)). Scale bar = 20 µm. Negative = negative control was performed without using the primary antibody.

**Figure 7:**
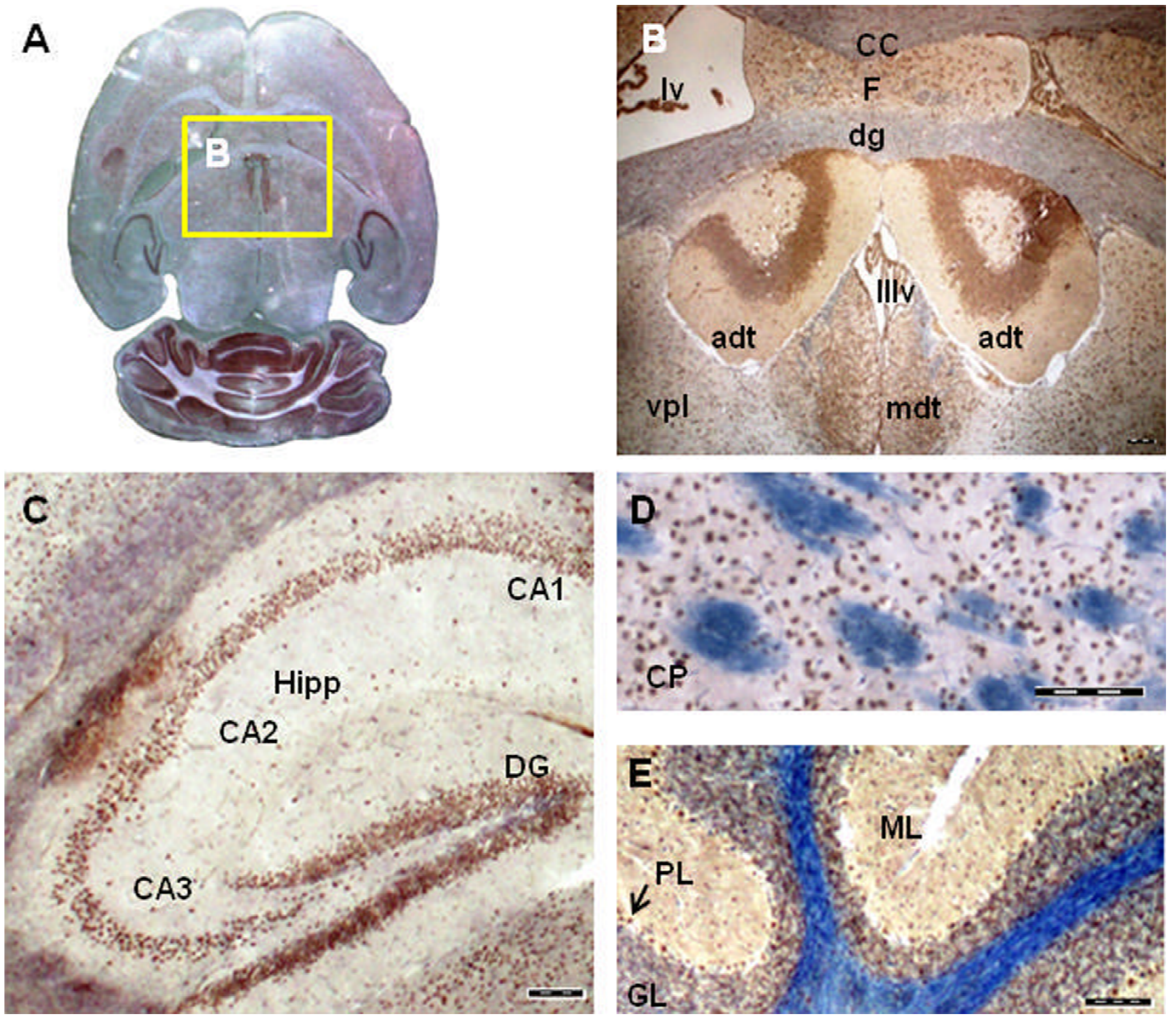
Fam124B expression in the mouse central nervous system. (**A**) Overview, (**B**) Thalamic nuclei, (**C**) Hippocampus, (**D**) Caudate Putamen, (**E**) Cerebellum. CC = Corpus Callosum, Hipp = Hippocampus, CA1-3 = Cornu Ammonis areas, DG = dentate gyrus, CP = Caudate Putamen, adt = anterior dorsal thalamic nucleus, dg = granual layer of dentate gyrus, lv = lateral ventricle, F = fornix, mdt = mediodorsal thalamic nucleus, IIIv = third ventricle with choroid plexus, vpl = ventral posterior thalamic nucleus, lateral part, ML = molecular layer of cerebellum, PL = purkinje cell layer of cerebellum, GL = granular layer of cerebellum, Scale bar = 100 µm.

**Figure 8:**
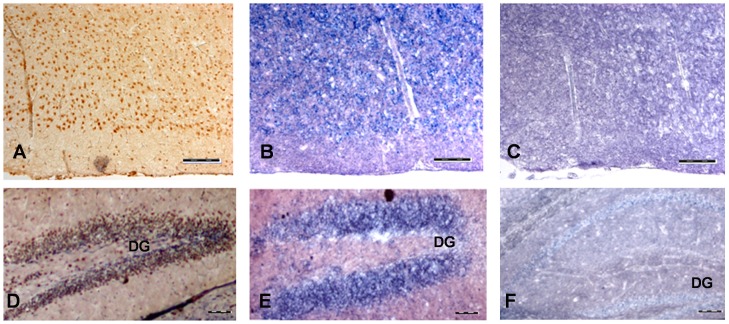
In situ hybridization of Fam124B on mouse brain cryosections in comparison with immunostaining. (**A**) Immunostaining of a cortex section, (**B**) In situ hybridization (ISH) of a cortex section (**C**) Sense control of ISH. (**D**) Immunostaining of a hippocampus section, (**E**) In situ hybridization of a hippocampus section (**F**) Sense control of ISH. DG = dentate gyrus. Scale bar = 100 µm.

The expression pattern of murine Fam124B was studied by semiquantitative reverse transcription polymerase chain reaction (RT-PCR) on RNA of wild type CD1 mouse tissues and E9.5 and E12.5 embryos (Figure 6A). The RT-PCR results were validated by quantitative real-time PCR (qRT PCR) in 3 biological and 3 technical replicates (data not shown). Relative mRNA expression levels were determined by using ΔCt values and were normalized to the housekeeping genes *Gapdh, Hprt* and *Sdna*. Although quantitative RT-PCR showed a high variability of the Fam124B expression status in different animals, the semiquantitative RT-PCR results could be confirmed, with highest expression rate in lung and lowest in liver. Western blotting and immunohistochemical staining (IHC) performed on adult mouse tissues confirmed the semiquantitative and quantitative RT-PCR results (Figure 6B and C). Furthermore, we evaluated the expression level of Fam124B in sections of adult mouse brains. Immunohistochemical examination demonstrated an expression of Fam124B in different brain areas (Figure 7). Fam124B is highly expressed in the cortex, the hippocampus subfields 1–3 (CA1-3), the dentate gyrus, the caudate putamen, and the cerebellum. In situ hybridization (ISH) of cortex and hippocampus sections with a full length Fam124B RNA probe supported the results obtained by IHC (Figure 8).

**Figure 9:**
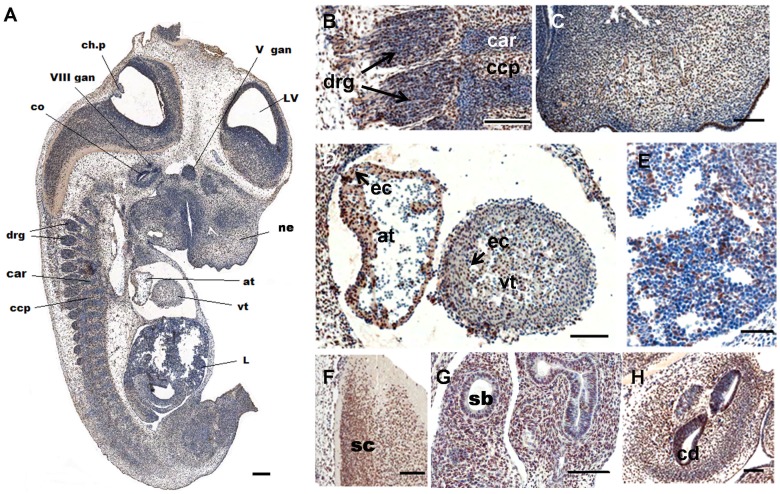
Fam124B expression at murine embryonic stage E12.5 (sagittal sections). Fam124B expression (brown) was found in a variety of embryonic tissues. Expression was observed in several brain areas, spinal cord, dorsal root ganglia, developing cochlea and surrounding tissues, lung, heart, and kidney. Low expression was found in the developing liver and no expression (blue) in blood cells. (**A**) Overview of Fam124B immunostaining on sagittal section of an E12.5 wildtype embryo slightly counterstained with haematoxylin, scale bar = 200 µm. Higher magnification of (**B**) Dorsal root ganglia, scale bar = 100 µm (**C**) Nasal region, scale bar = 100 µm (**D**) The developing heart, scale bar = 100 µm (**E**) The developing liver (L), scale bar = 50 µm (**F**) spinal cord (sc), scale bar = 50 µm (**G**) The developing lung with segmental bronchus (sb), scale bar = 100 µm (**H**) Cochlea, and surrounding tissue, scale bar = 100 µm. LV = lateral ventricle, ch.p = choroid plexus differentiating from fourth ventricle, co = cochlea, cd = cochlear duct, ne = nasal epithelium, V gan = left trigeminal (V) ganglion, drg = dorsal root ganglion, VIII gan = Vestibulocochlear (VIII) ganglion, at = left atrium of heart, vt = left heart ventricle, ec = endothelial cells, car = cartilage primordium of body of vertebra, ccp = cartilage condensation being primordium of vertebral body.

To explore the expression pattern of Fam124B during mouse development, we extended our IHC studies to sections of E12.5 mouse embryos, the time point when organogenesis takes place. Similar to adult mouse tissues, Fam124B expression at E12.5 was observed in different inner organs, with lowest expression in liver tissue (Figure 9A–H). In the developing heart, high expression was detected particularly in the endothelial cells of the atrium and along the trabeculated endocardium of the ventricle, whereas no expression was observed in blood cells (Figure 9D). At E12.5 when the developing lung is not yet divided into lobes, Fam124B expression was detected in the stroma cells and in the epithelial cells of the segmental bronchi (Figure 9G). Furthermore, Fam124B was expressed in the developing cochlea and the surrounding tissue (Figure 9E) as well as in neural cells. The dorsal root ganglia and the precartilage condensation zones in the neural arch showed Fam124B expression, while Fam124B expression was significantly reduced in the cartilage (Figure 9B). High Fam124B expression could be found in the spinal cord (Figure 9F) and in the developing brain (Figure S1).

## Discussion

Loss of function mutations in *CHD7* lead to CHARGE syndrome, a well known malformation syndrome affecting several organs and sensory systems. CHD7 regulates the transcription of tissue specific target genes through the mechanism of ATP-dependent chromatin remodeling [9], [12], [13], [7]. Chromatin remodeling proteins are typically found in large multiprotein complexes. For CHD7, several tissue specific interacting partners are described [8], [10], [12], [13]. Thus, it can be suggested that there are cell type and developmental stage specific compositions of CHD7 containing complexes. However, the precise complex compositions for each tissue are still unknown. In an earlier study, we demonstrated that a part of human CHD7 interacts with a part of human CHD8 both directly and indirectly, via potential linker proteins [11]. Loss of function mutations in *CHD8* as well as de novo missense mutations were described in autism spectrum (ASD) and neurodevelopmental (NDD) disorder patients [19]–[22]. Interestingly, Betancur and colleagues described autism spectrum disorders in about two thirds of children with CHARGE syndrome [25], which supports our hypothesis of a CHD7-CHD8 containing complex regulating the same cell type specific target genes.

The identification and characterization of associated factors of a CHD7-CHD8 containing complex might play an important role in understanding the pathophysiology of CHARGE syndrome and ASD/NDD. Therefore, to further characterize this complex and to identify additional associated polypeptides interacting with the recently described human CHD7 and CHD8 part, we used the method of stable isotope labeling by amino acids in cell culture (SILAC) in combination with mass spectrometry. As a result of three biological replicates we identified the hitherto uncharacterized protein FAM124B as a potential interacting partner. In humans, two FAM124B transcript variants are present, while in mice, only one transcript, homologous to the human transcript variant 1, exists. The interaction of both human FAM124B transcript variants with the CHD7 and CHD8 part was confirmed by co-immunoprecipitation experiments. Direct yeast-two hybrid studies were performed to specify the FAM124B - CHD7 - CHD8 interaction areas. Both transcripts of human FAM124B interact directly with the CHD8 part containing the amino acids 1789–2302 (NP_065971.2), while the FAM124B - CHD7 interaction is an indirect interaction or the interacting area is outside of the used CHD7 part spanning amino acids 1591-2181 (NP_060250.2). The biological role of human FAM124B or its orthologs in *P.troglodytes, C.lupus, B.taurus, R.norvegicus, M.musculus, G.gallus, D.rerio* is still unknown. Bioinformatic analysis of the amino acid sequence failed to identify any known functional domain. To gain further insight into the biological role of FAM124B and its mouse ortholog, we examined the subcellular localization and the expression pattern. Immunofluorescence microscopy of overexpressed FAM124B indicated that overexpressed FAM124B is localized in the nucleus. Using the FAM124B antibody we could observe in untransfected HeLa cells a nuclear, but weaker signal as well, possibly indicating a lower expression of endogenous FAM124B. CHD8 is described as a nuclear protein as well [26]. Concerning CHD7, a dual localization in the nucleoplasm and in the nucleolus was observed [13]. Therefore, the interaction between FAM124B, CHD8 and CHD7 is supposed to take place in the nucleus.

We detected Fam124B expression in various adult mouse tissues, with highest expression in lung and heart, followed by kidney, brain and testis, whereas its expression was weakest in liver tissue. No Fam124B expression could be observed in blood cells. In adult mouse brain sections, we demonstrated Fam124B expression in several areas. Within the brain, high expression levels were found in the cortex, granular and purkinje cell layer of the cerebellum, thalamic nuclei, caudate putamen, and hippocampus.

CHD8 expression was previously noted in various adult mouse tissues including heart, brain, spleen, lung, liver, skeletal muscle, kidney and testis [26].

In adult mouse tissues, CHD7 expression was determined in the retina, in the cornea, brain, skeletal muscle, heart, kidney, lung, olfactory epithelium and olfactory bulb [1], [27]. According to these previously described data we observed an overlapping expression pattern between CHD7, Chd8 and Fam124B in adult mouse tissues. Due to the fact that CHARGE syndrome is a developmental disorder and NDD/ASD are caused by abnormal brain development, we extended our Fam124B expression studies to embryonic tissues. The expression pattern of CHD7 during development has been studied in embryos of different mammalian species, before [28], [2], [5], [29], [27], [30]. During mouse development, Chd7 was found to be expressed at E12.5 in a wide range of head tissues (several brain areas with highest expression in proximity to the ventricles, in the choroid plexus, developing olfactory epithelium, ganglia of the cranial nerves, otic and optic pits, and the developing inner ear) and several regions of the body (especially the dorsal root ganglia and lung epithelium, as well as stomach epithelium, kidney and heart) [28]–[30]. Fam124B expression at E12.5 was found in a variety of embryonic tissues (e.g. several brain areas, spinal cord, dorsal root ganglia, developing cochlea and surrounding tissues, lung, heart, and kidney), as well. Similar to the results of adult mouse tissues, Fam124B expression at E12.5 correlated in many embryonic tissues with the Chd7 expression pattern, and therefore Fam124B was found to be expressed in organs affected in CHARGE syndrome.

It has already been shown that CHD7 binds to methylated histone H3 lysine 4 (H3K4) at enhancer elements and modifies cell type specific gene expression in a fine-tuning manner [9], [12]. Schnetz and colleagues hypothesized that the binding of a CHD7 containing complex to enhancer elements may modulate the expression rate of target genes through enhancer-promotor interactions [9], [12]. In mouse ES cells, a subset of Chd7 sites colocalize with p300, Oct4, Sox2 and Nanog. The subset of Chd7 sites which are not co-occupied by these proteins can also enhance transcription [12]. However, the associated factors of these Chd7 sites are still unknown. Possibly, the identified novel Fam124B may serve as an associated factor involved in Chd7 enhancer-mediated transcription. Furthermore, the high Fam124B expression in the developing mouse brain and in neuronal tissues at embryonic day E12.5 might indicate a role of FAM124B together with CHD8 in neurodevelopmental and autism spectrum disorders.

In summary, we identified FAM124B as a binding partner of a part of CHD7 and CHD8. We conclude that FAM124B is an associated factor of a CHD7 and CHD8 containing complex. Fam124B is widely expressed at mouse developmental stage E12.5 with an overlapping expression to Chd7 and high expression in the developing mouse brain. Therefore, we assume a role for FAM124B in the pathogenesis of CHARGE syndrome and NDD/ASD. The results of our interaction studies, the subcellular localization, and expression profile of FAM124B provide valuable information and represent a starting point for further functional investigations on FAM124B and its possible role in CHARGE syndrome and NDD/ASD.

## Materials and Methods

### Ethics Statement/mouse Strains

The animal studies were approved by the Institutional Animal Care and Use Committee of the University of Göttingen. All mouse studies were performed on CD1 wildtype mice.

### Stable Isotope Labeling by Amino Acids in Cell Culture (SILAC) and Mass Spectrometry (MS)

Two HeLa cell populations were grown in SILAC DMEM culture media (10% dialyzed FBS and 1% penstrep) (PAA Laboratories GmbH, Pasching, Austria; Thermo Fisher Scientific, Waltham, MA). The “*light”* SILAC medium was supplemented with unlabeled l-lysine and l-arginine, while the “heavy” SILAC medium was supplemented with arginine and lysine containing heavy isotopes of carbon and nitrogen (^13^C_6_-lysine and ^13^C_6_^15^N_4_-arginine). Upon metabolic labeling of the cells this leads to a mass shift of +6 and +10 Da per incorporated arginine and lysine, respectively. After at least 5 cell passages, co-transfection with the previously described plasmids CHD7-CR1-3-pCMV-HA and CHD8-pCMV-cmyc [11] into either L- or H-labeled HeLa cells was performed. Non transfected L- or H-labeled cells were used as negative control. After transient co-transfection, the cells were incubated at 37°C for 24 hours, then washed with DPBS (PAN BIOTECH GmbH, Aidenbach, Germany) and trypsinized (Invitrogen, Carlsbad, CA, USA). To isolate the same amount of protein from both cell cultures, cell populations were accurately counted under microscope using counting chambers (Neubauer haemocytometer) (approximately 1 - 1,2×107 cells were used) before cell lysis, which was performed as described by Oellerich et al. (2011) [24]. Protein lysates were incubated with 4 µg of monoclonal anti-Ha antibody (Roche Diagnostik GmbH, Mannheim, Germany) at 4°C overnight. Proteins coupled with the antibody were incubated at 4°C with 100 µl of protein G (Immunoprecipitation Kit, Roche Diagnostik GmbH, Mannheim, Germany) for further 2 hours. Centrifugation at 350 rpm for 3 min was carried out to collect the agarose beads. To remove unbound proteins, the beads were washed two times in 500 ml lysis buffer and one time with 1 ml washing buffer 2 (Immunoprecipitation Kit, Roche Diagnostik GmbH, Mannheim, Germany). The beads were resuspended in 50 µl NuPAGE LDS Sample buffer (Invitrogen, Carlsbad, CA, USA) supplemented with 0.1 M DTT. After denaturation for 10 min at 95°C, the denaturated H- and L-labeled proteins were pooled in equimolar amounts and subsequently the samples were separated by size using an 1D-PAGE (Invitrogen, Carlsbad, CA, USA) followed by coomassie blue staining. Each gel lane was cut into 23 gel slices. Proteins from each slice were in-gel digested with trypsin (Promega, Madison, WI) according to the protocol described by Shevchenko et al. (2006) [31]. MS measurements were carried out using a nanoflow HPLC (Agilent, Boeblingen, Germany) coupled to a nanoelectrospray LTQ-Orbitrap XL mass spectrometer (Thermo Fisher Scientific, Waltham, MA) as described by Nikolov et al. (2011) [32].

### RNA Isolation and Semiquantitative Reverse-transcription PCR

Total RNA was isolated from HeLa cells, MRC-CV1 cells or different mouse tissues using Trizol reagent (Invitrogen, Carlsbad, CA, USA). Homogenizing was carried out with Tissue lyser LT (Qiagen GmbH, Hilden, Germany). 5 µg of total RNA from each sample was reverse-transcribed using Superscript II reverse transcriptase (Invitrogen, Carlsbad, CA, USA). For each PCR reaction 0.5 µl undiluted cDNA and 0.2 µl platinum taq polymerase (Invitrogen, Carlsbad, CA, USA) was used. Human cDNA isolated from HeLa and MRC-CV1 cells were amplified using the following primers: 5′-CCTTCTACACACGCTGTCTTTG-3′ as forward primer and 5′-GGGGATGAGCTATGCAACCTAAG-3′ as reverse primer. RT-PCR on mouse tissues was carried out using the following primer pair: forward primer 5′-GGTGGCCTTCATCATAATCTTC-3′ and reverse primer 5′-CCAGTCACACTCTTGCT TCTGT-3′. The correct sequence was confirmed by sequence analysis.

### Quantitative RT-PCR

Realtime PCR reactions were performed using 1 µl of 1∶10 diluted cDNA from different CD1 mouse tissues, SYBR green (Invitrogen, Carlsbad, CA, USA) and the gene-specific primers forward 5′-CCGTGTGTTCCCATCAGCAG-3′ and reverse 5′- CTCCTCCTCCGGCTCCTTG-3′. Relative mRNA expression levels were determined using ΔCt values and were normalized to the housekeeping genes *Gapdh, Hprt* and *Sdna*. The experiment was performed in three biological and 3 technical replicates.

### Co-immunoprecipitation

For co-immunoprecipitation studies we generated the plasmids FAM124B-1,3-pCMV-HA (transcript variant 1, NP_001116251.1), FAM124B-1,2-pCMV-HA (transcript variant 2, NP_079061.2), FAM124B-1,3-pCMV-cmyc (transcript variant 1, NP_001116251.1) and FAM124B-1,2-pCMV-cmyc (transcript variant 2, NP_079061.2) by cloning the human full-length sequence of transcript variant 1 and transcript variant 2 in frame to the hemagglutinin (HA) epitope tag into the pCMV-HA vector (Clontech, Mountain View, CA, USA) or in frame to the N-terminal c-Myc epitope tag into the pCMV-Myc vector (Clontech, Mountain View, CA, USA) by using the In Fusion Advantage Kit (Clontech, Mountain View, CA, USA) according to the company’s protocol. The correct reading frame and sequence were confirmed by sequence analysis.

HeLa cells were cultured in 50 ml flasks (Sarstedt, Newton, NC, USA) and were co-transfected with CHD7-CR1-3-pCMV-HA (amino acids 1593-2178, NP_060250.2) and either FAM124B-1,3-pCMV-cmyc or FAM124B-1,2-pCMV-cmyc. Additionally, we co-transfected HeLa cells with CHD8-pCMV-cmyc (amino acids 1789-2302, NP_065971.2) and either FAM124B-1,3-pCMV-HA or FAM124B-1,2-pCMV-HA. After 24 h of incubation at 37°C, co-immunoprecipitations were carried out as previously described [11]. For immunoblotting we used the following antibodies: anti-c-Myc (abcam, ab9106) at a dilution of 1∶2000, anti-HA (Roche Diagnostik GmbH, Mannheim, Germany) at a dilution of 1∶1000, goat anti-CHD7 (abcam, ab 65097) at a dilution of 1∶2000, rabbit anti-CHD8 (abcam, ab84527) at a dilution of 1∶2000. The following antibodies were used for detection: goat anti-rabbit IgG peroxidase secondary antibody (Sigma-aldrich, St.Louis, MO, USA) for anti-c-Myc and anti-CHD8, goat anti-Rat secondary antibody conjugated with horseradish peroxidase (Thermo Scientific, Rockford, IL, USA) for anti-HA, donkey anti-goat IgG peroxidase secondary antibody (Santa-Cruz Biotechnologies, California, USA) for anti-CHD7.

### Yeast Two Hybrid

For yeast two hybrid studies we generated the constructs FAM124B-1,3-pGBKT7 (transcript variant 1, NP_001116251.1), FAM124B-1,2-pGBKT7 (transcript variant 2, NP_079061.2), FAM124B-1,3-pGADT7 (transcript variant 1, NP_001116251.1) and FAM124B-1,2-pGADT7 (transcript variant 2, NP_079061.2). The human full length sequence of transcript variant 1 and transcript variant 2 were subcloned in frame to the ATG of the pGBKT7 or pGADT7 vector (Clontech, Mountain View, CA) by using the In Fusion Advantage Kit (Clontech, Mountain View, CA). All constructs were transformed into Y2HGold strain cells (Clontech, Mountain View, CA) and tested for toxicity and autoactivation of the bait or prey reporter genes. No toxicity for the yeast cells could be observed, but the FAM124B-1,3-pGBKT7 and FAM124B-1,2-pGBKT7 showed autoactivation. Therefore, the further experiments were performed with the FAM124B-1,3-pGADT7 and FAM124B-1,2-pGADT7 constructs. These both plasmids were co-transformed into Y2HGold strain cells together with either the CHD7-CR1-3-pGBKT7 (amino acids 1591-2181, NP_060250.2) or the CHD8-pGBKT7 (amino acids 1789–2302, NP_065971.2) plasmids. The direct Yeast two hybrid was performed according to the manufactures’ protocol.

### Cryosections

Adult brains from CD1 mice were isolated and fixed overnight in 4% paraformaldehyde (PFA) solution prepared in sterile PBS with DEPC water at 4°C with gentle shaking. On the next day, the tissues were washed 3 times with sterile PBS/DEPC at 4°C and incubated in autoclaved/sterile 30% sucrose at 4°C with gentle shaking till the tissues sank down. Then the brains were incubated for 30 minutes to 1 hour at 4°C in a 1∶1 mixture containing 30% sucrose and Jung tissue freezing medium (Leica Microsystems, Nussloch, Germany), following an incubation in tissue freezing medium at 4°C for 30 minutes and embedded on dry ice in tissue freezing medium. The frozen block was kept till usage at −80°C and cut by cryo-microtome (Leica Instrument, Nussloch, Germany) with 20 µm thickness and sticked on Menzel Superfrost microscope slides (Thermo Scientific, Rockford, IL, USA).

### In situ Hybridization

In situ hybridization was performed on cryosections of adult brains from CD1 mice. The following primer pairs were used to amplify by RT-PCR the whole coding region of *Fam124B*: forward 5′- GCCATGGATGAGATACAGGAA-3′ and reverse 5′- CATGAATGGGGCTGACTCTTA-3′. The PCR product was subcloned into the pGEMT easy vector (Promega, Madison, WI) and transformed into *E.coli*. Positive plasmids were isolated and sequenced. For probe amplification from a correct plasmid the following primers were used for the Sp6 transcript: pGEM-T-Sp6-F (ACGTCGCATGCTCCCG) and pGEM-T-R2 (CCAGGCTTTACACTTTATGCTTCC) and for the T7 transcript: PCR-T7 (CTGCGCAACTGTTGGG) and pGEM-T-R1 (AGGCGGCCGCGAATTCAC). The PCR products were gelextracted and DIG-labelled RNA probes were synthesized using the DIG RNA Labeling Kit (SP6/T7) (Roche Diagnostik GmbH, Mannheim, Germany) according to the manufacturer’s protocol. Synthesized probes were ethanol precipitated and resuspended in 100 µl of DEPC water/formamide 1∶1 mixture and kept till usage at −20°C. Non-radioactive in situ hybridization have been done according to Moorman et al. (2001) [33]. The results have been observed using a BX60 microscope (Olympus, Hamburg, Germany) and images were processed by the analysis program (CellSens Dimension, Olympus).

### Immunocytochemistry on HeLa Cells

HeLa cells were transfected with the FAM124B-1,3-pCMV-cmyc construct. Cell culture and staining procedure was carried out as described previously [11]. As a blocking solution 10% sheep serum diluted in DPBS with 0.1% Tween 20 (TPBS) was used. Rabbit anti-FAM124B antibody (Proteintech Group, Chicago, IL) was diluted at 1∶50. As secondary antibody anti-rabbit IgG conjugated with Cy3 (Sigma-Aldrich, St.Louis, MO, USA) antibody and anti-mouse IgG conjugated with FITC (Sigma-aldrich, St.Louis, MO, USA) antibody were used at a dilution of 1∶400. Imaging was performed and processed using Olympus Fluoview FV1000 microscope (Olympus, Hamburg, Germany) and images were analyzed using the program FV10-ASW version 01.07.03.00.

### Isolation of Protein and Immunoblotting

Total fractions of proteins were isolated from adult tissues of CD1 mouse line using RIPA buffer (50 mM Tris-HCl pH 74, 1% NP-40, 0.25% Sodium-deoxycholate buffer, 150 mM NaCl, 1 mM EDTA and protease inhibitors). Proteins were separated by a 4–12% NuPAGE Bis-Tris gel (Invitrogen, Carlsbad, CA, USA) and transferred to a nitrocellulose membrane (0.45 µm) using a semi-dry system at 100 mA for 90 minutes. Membranes were blocked with 5% BSA/TBST (PAA laboratories, Linz, Austria) for Fam124B blotting while 5% milk/TBST (Carl Roth GmbH, Karlsruhe) blocking buffer was used for all other blottings. The blocked membranes were incubated with appropriate primary antibodies overnight at 4°C. Rabbit anti-FAM124B (Proteintech Group, Chicago, IL) was diluted at 1∶200 in 1% BSA/TBST, rabbit anti-c-Myc (abcam, ab9106) and mouse anti-HSC70 (sc-7298, Santa Cruz Biotechnology, California, USA) were diluted at 1∶2000 and 1∶10000 in 2% milk/TBST, respectively. After washing, the membrane was incubated with the following secondary antibodies: anti-rabbit IgG peroxidase secondary antibody (Sigma-aldrich, St.Louis, MO, USA) for anti-c-Myc and anti-Fam124B, anti-mouse IgG peroxidase secondary antibody (Jackson ImmunoResearch Laboratories, Pennsylvania) for anti-HSC70. The membrane was washed and super signal West Pico chemiluminescent substrate (Thermo Scientific, Rockford, IL, USA) was applied on the membrane and positive bands were detected on a clear blue X-ray film (Thermo Scientific, Rockford, IL, USA).

The nuclear fraction of untransfected HeLa cells and FAM124B-1,3-pCMV-cmyc overexpressed HeLa total cell lysate was immunoblotted using anti-FAM124B (Proteintech Group, Chicago, IL) at a dilution of 1∶200. FAM124B-1,3-pCMV-cmyc overexpressed HeLa total cell lysate was immunoblotted additionally with anti-c-Myc (abcam, ab9106) at a dilution of 1∶2000. Protein quality was controlled using mouse anti-HSC70 (sc-7298, Santa Cruz Biotechnology, California, USA) at a dilution of 1∶10000.

### Immunohistochemistry on Paraffin Embedded Tissues

Adult wild type CD1 mouse tissues and E12.5 embryos were paraffin embedded according to standard procedures. From the paraffin embedded tissues 7 µm sections were made and incubated overnight at 45°C. After a three time xylene incubation (10, 5, 5 minutes) a series of ethanol incubation (100%, 95%, 90%, 80%, 70%, 50%) and a washing step with DPBS for 2 minutes followed. Slides were cooked with an antigen retrieval buffer (citric acid 0.1 M; EDTA 0.01%, pH 6.2) in a steam cooker for 10 minutes, followed by a cool down step on ice for at least 10 minutes. Endogenous peroxidase activity was blocked using 6% H_2_O_2_ solution in water for 15 minutes. The solution was tapped off from the slides and blocked with horse serum (Vectastain Universal Quick kit, Vector Laboratories, Burlingame, CA) for 30 minutes in a humidity chamber at room temperature. Rabbit anti-FAM124B (Protein Tech), rabbit anti-CHD7 (abcam, ab31824) and rabbit anti-CHD8 (abcam, ab84527) antibodies were diluted at 1∶50, 1∶50 and 1∶100, respectively. The primary antibodies were incubated at 4°C overnight. After washing with TPBS for 5 minutes, a Pan specific secondary antibody (Vectastain Universal Quick kit, Vector Laboratories, Burlingame, CA) was applied for maximum 10 minutes and washed again with TPBS for 5 minutes. Streptavidin peroxidase (Vectastain Universal Quick kit, Vector Laboratories, Burlingame, CA) was applied on the slides for 5 minutes. Slides were then transferred to TPBS washing buffer for minimum 5 minutes. The staining was developed with DAB substrate (Roche Diagnostik GmbH, Mannheim, Germany) for around 2 minutes. The slides were rinsed for minimum 5 minutes under tap water and counterstained in mayor’s hematoxylin for 20–40 seconds and washed with tap water 3–4 times. Mounting of slides were done with Aqua polymount (Polysciences Inc., Warrington, PA). Imaging was performed and processed using a BX60 microscope (Olympus, Hamburg, Germany) and the analySIS program Cell-Sens Dimension.

## Supporting Information

Figure S1 **Fam124B expression in the developing brain at murine embryonic stage E12.5 (coronal sections). (A)** Overview of Fam124B immunostaining of the developing brain slightly counterstained with haematoxylin. **(B)** Higher magnification of the trigeminal ganglion, **(C)** Aqueduct of sylvius with choroid plexus = AQ **(D)** Higher magnification of the medulla (me) with spinal tract of trigeminal nerve (Vsp), nV = trigeminal nucleus, MLF = medial longitudinal fasciculus **(E)** Higher magnification of the eye, nr = neural retina, lf = lens fibers, vb = vitreous body. Scale bar = 50 µm.
(TIF)

Table S1 **Proteins found by SILAC in combination with mass spectrometry 1^st^ experiment (XLS).**
(XLS)

Table S2 **Proteins found by SILAC in combination with mass spectrometry 2^nd^ experiment (XLS).**
(XLS)

Table S3 **Proteins found by SILAC in combination with mass spectrometry 3^rd^ experiment (XLS).**
(XLS)
