# Supplementary figures and images for: Identification and Characterization of FAM124B as a Novel Component of a CHD7 and CHD8 Containing Complex
Source: PLoS One. 2012 Dec 21;7(12):e52640. doi: 10.1371/journal.pone.0052640 (PMC3528654; doi:10.1371/journal.pone.0052640)

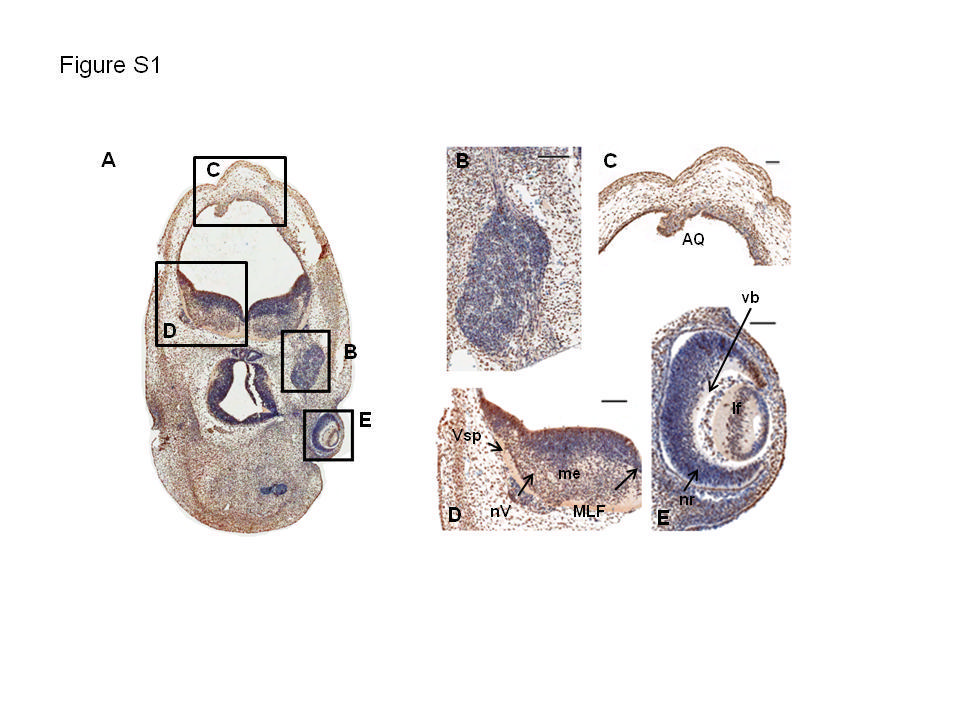

Supplement: Figure S1 — Fam124B expression in the developing brain at murine embryonic stage E12.5 (coronal sections). (A) Overview of Fam124B immunostaining of the developing brain slightly counterstained with haematoxylin. (B) Higher magnification of the trigeminal ganglion, (C) Aqueduct of sylvius with choroid plexus = AQ (D) Higher magnification of the medulla (me) with spinal tract of trigeminal nerve (Vsp), nV = trigeminal nucleus, MLF = medial longitudinal fasciculus (E) Higher magnification of the eye, nr = neural retina, lf = lens fibers, vb = vitreous body. Scale bar = 50 µm. (TIF) [file pone.0052640.s001.tif]
